# Factors Influencing Doctors’ Participation in the Provision of Medical Services Through Crowdsourced Health Care Information Websites: Elaboration-Likelihood Perspective Study

**DOI:** 10.2196/16704

**Published:** 2020-06-29

**Authors:** Yan Si, Hong Wu, Qing Liu

**Affiliations:** 1 School of Business, Wuxi Vocational College of Science and Technology Wuxi China; 2 School of Medicine and Health Management Tongji Medical College Huazhong University of Science and Technology Wuhan China

**Keywords:** crowdsourcing, crowdsourced medical services, online health communities, doctors’ participation, elaboration-likelihood model

## Abstract

**Background:**

Web-based crowdsourcing promotes the goals achieved effectively by gaining solutions from public groups via the internet, and it has gained extensive attention in both business and academia. As a new mode of sourcing, crowdsourcing has been proven to improve efficiency, quality, and diversity of tasks. However, little attention has been given to crowdsourcing in the health sector.

**Objective:**

Crowdsourced health care information websites enable patients to post their questions in the question pool, which is accessible to all doctors, and the patients wait for doctors to respond to their questions. Since the sustainable development of crowdsourced health care information websites depends on the participation of the doctors, we aimed to investigate the factors influencing doctors’ participation in providing health care information in these websites from the perspective of the elaboration-likelihood model.

**Methods:**

We collected 1524 questions with complete patient-doctor interaction processes from an online health community in China to test all the hypotheses. We divided the doctors into 2 groups based on the sequence of the answers: (1) doctor who answered the patient’s question first and (2) the doctors who answered that question after the doctor who answered first. All analyses were conducted using the ordinary least squares method.

**Results:**

First, the ability of the doctor who first answered the health-related question was found to positively influence the participation of the following doctors who answered after the first doctor responded to the question (β_offline1_=.177, *P*<.001; _βoffline2_=.063, *P*=.048; β_online_=.418, *P*<.001). Second, the reward that the patient offered for the best answer showed a positive effect on doctors’ participation (β=.019, *P*<.001). Third, the question’s complexity was found to positively moderate the relationships between the ability of the first doctor who answered and the participation of the following doctors (β=.186, *P*=.05) and to mitigate the effect between the reward and the participation of the following doctors (β=–.003, *P*=.10).

**Conclusions:**

This study has both theoretical and practical contributions. Online health community managers can build effective incentive mechanisms to encourage highly competent doctors to participate in the provision of medical services in crowdsourced health care information websites and they can increase the reward incentives for each question to increase the participation of the doctors.

## Introduction

### Background

The imbalance between the supply and demand for medical services has caused conflicts in the patient-doctor relationship, especially because the health awareness of patients has dramatically increased in recent years [1]. With the development of online health communities in China, an increasing number of people have begun to seek web-based health information and services [2,3], and these websites have become a useful complementation [4]. However, only 6.1% of the doctors participate in online health communities to provide medical services [5]. Medical services are not easily accessible for patients in China [6], especially for patients with serious diseases and for those living in remote areas [7]. The improvement of doctors’ participation in online health communities is the key to enhancing timely services and supplementary services, which will reduce the conflicts in the patient-doctor relationship and eventually improve the overall health of the country [8]. Therefore, the primary concern of the governments and health care organizations is to increase the number of doctors involved in the provision of web-based medical services.

Crowdsourcing is widely used among organizations to obtain more and better solutions for their projects by encouraging the public to perform tasks by sharing their knowledge and skills together [9,10]. It is an emerging organizational practice that has attracted much attention over the last decade, and this pattern has also emerged in the health care field [7,11,12]. Crowdsourcing is a mode of engaging a crowd of people to achieve a common goal, for example, for solving problems by sharing the problem through questionnaires and then considering the responses of all the people in the network [13-15]. In crowdsourcing, a wide range of goals can be achieved—from idea gathering to solution elaboration [16]. Crowdsourcing is also used to survey infectious diseases by capturing the symptom data that has been submitted voluntarily [17,18]. With the rapid development of the internet, an increasing number of medical question-and-answer websites have adopted the crowdsourcing mode to find better answers to solve patients’ health problems, such as *Medhelp.org* in the United States and *120ask.com* in China. These crowdsourced health care information websites are widely accepted by patients [19]. This service is a type of expert-based crowdsourced medical service [20,21], which allows patients to post an “open-call” question to undefined doctors [22] with relatively low cost [16,23]. Crowdsourced health care information websites have adopted an active crowdsourcing mode, that is, the patient has an active role, wherein he/she poses a particular medical question and solicits relevant information, knowledge, opinion, and ideas from doctors [24]. By using the crowdsourced health care information websites, patients hope to describe the symptoms and receive the diagnosis and treatment of diseases and be prescribed drugs, similar to that received in common medical services. Moreover, the patients expect that doctors who play vital roles in such services will offer answers to their questions. The most apparent feature of crowdsourced health care information websites is that more than one doctor can give answers, based on their knowledge and experience, to the same question from a single patient. Therefore, by using crowdsourced health care information websites, patients can obtain more comprehensive and better suggestions.

Previous studies have investigated the motivations behind the behavior of the participating users in posting their ideas on crowdsourcing websites [25-28]. These motivations can be divided into 2 dimensions: extrinsic motivations [25-27] and intrinsic motivations [28-30]. For the extrinsic motivations, researchers have shown that financial incentives such as monetary stimulus play an important role in the users’ participating behaviors [31]. Some studies have shown that the reward is the primary source of income on the crowdsourcing platforms and this reward drives users to participate in tasks [25-27]. For intrinsic motivations, some studies have proposed that the reasons for participation in crowdsourced tasks include factors such as competency, reputation, altruism, and learning, which are the critical driving forces of the participation behaviors [28-30]. The number of downloads means attention is the motivation for users to participate in YouTube [32]. However, previous studies have mainly focused on the users’ participating behaviors in other products or service fields, and only little attention has been paid to the users’ participating behaviors in the medical field and in empirical research from the perspective of the information system.

We employed the elaboration-likelihood model (ELM) as the theoretical base to understand how doctors process information regarding participation in the provision of medical services in crowdsourced health care information websites. The ELM originates from social psychology and argues that individuals can change their attitudes through a dual route, namely, the central route and the peripheral route [33]. In the “central route,” an individual processes information such as information quality and content through careful in-depth thinking. On the contrary, in the “peripheral route,” the individual makes a decision based on less cognitive thinking and simple information cues such as monetary value [34,35]. The ELM is a dual-process theory arguing that persuasion can act via the central or the peripheral route, and it is the process of the individual’s attitude change as a result of being influenced by the mental effort required for the message [33,36]. The ELM also indicates that the dual routes of decision making are moderated by the potential user’s motivation to elaborate on informational messages [33,36]. Since the sustainable development of crowdsourced health care information websites depends on doctors’ participation, we aimed to investigate the factors influencing the doctors’ participation in providing health care information on these crowdsourced websites from the elaboration-likelihood perspective. The research questions were as follows.

1. What factors affect doctors’ participation in crowdsourced health care information websites?
2. How can the question’s complexity moderate the central route and peripheral route?

### Research Framework and Hypotheses Development

Based on the framework of ELM, this study aims to investigate the attitude of the participating doctors toward the crowdsourced health care information websites, which is persuaded by dual-process cues, namely, central cues and peripheral cues.

#### Central Cues

Based on the ELM framework, central cues information is a signal of project quality, which has significant positive effects on the recipient’s choice [37,38]. Information quality and review quality of products or service providers are often regarded as the central cues [39-41]. The purchase behaviors of the consumers are also considered as an important signal of the product or service quality that attract other consumers to follow and make decisions [42,43]. The reputation, ability, purchase behaviors, and review behaviors of consumers can influence the decisions of the following consumers [44]. Therefore, we hypothesize that the behaviors of the doctors who answered first would be a signal to other doctors and this could influence their behaviors. Specifically, the ability of the first doctor who answered might convey a signal of the question information, that is, the question is considered worthy of answering if the reputation/ability/review rating of the doctor who answered first is high. The doctors are free to answer any question of the patients in the question pool in these crowdsourced websites. Doctors can obtain information on the questions and the former answers, including title and reputation, especially of the doctor who answered the question first. Therefore, the following doctors’ participation would be influenced by information about the doctor who answered first. The ability of the doctor who answered the patient’s question first is especially crucial as the quality of the doctor’s answer is considered very important in an empirical model. We hypothesize that questions that are answered first by highly competent doctors will gain more attention from other doctors, and the other doctors will be driven to participate in the provision of medical services through these crowdsourced health care information websites. Thus, our first hypothesis was called the central route hypothesis and it was as follows: The ability of the doctor who answered first has a positive effect on the following doctors’ participation in crowdsourced health care information websites.

#### Peripheral Cues

Peripheral cues are information based on less cognitive effort such as the numbers or source characteristics that rely on shortcuts [37,45]. The reward is monetary numbers, which accord with the peripheral cue. Previous studies have explored the role of reward in the crowdsourcing field [46-48] and have indicated that financial reward is the most critical motivation, as most respondents reported that they do not perform tasks for fun or to kill time [31]. Some studies have shown that money or points have a positive effect on the user’s participation in online health communities [28,49]. Further, the effects of monetary incentives on other specified crowdsourcing tasks were studied [50-53]. Thus, the participation behavior of the doctors is influenced by the monetary reward, which is listed on the question information. We believe that doctors would tend to answer questions with higher expected rewards. Thus, our second hypothesis was called the peripheral cue hypothesis, which is as follows: The reward provided by the patient has a positive effect on the following doctors’ participation in crowdsourced health care information websites.

#### Moderating Effects

The following hypotheses are based on the moderating effects of the question’s complexity on the central route and the peripheral route. The elaboration of the moderator will positively moderate the influence of the central route and negatively moderate the influence of the peripheral route [54]. Based on the ELM framework, the use of the central route and peripheral route processing for decision making is moderated by the user’s ability and motivation to elaborate on informational messages [55-57], and motivation levels change the likelihood of elaboration by a user [56,57]. Patients can search/ask for information on their health problems and disease symptoms and find/ask information on the medications or other medical and health-related information in crowdsourced health care information websites. Patients with severe illness may ask more complex questions to doctors. In addition, highly complex questions can arouse the attention of doctors who have higher competencies than other doctors in clinical settings or in web-based medical services. Therefore, the solutions for highly complex questions rely more on the doctors’ competency. In addition, the reward that a patient assigns is often lower than that assigned in a normal web-based health service [58]; therefore, the behavior of answering health-related questions is an act of altruism, which means that the doctor provides an answer for the “public good” [59] of the patients or health-information seekers. We believe that doctors would take the effort to solve a health problem as an act of kindness rather than for money when the question complexity is high. We hypothesized that the question’s complexity has a moderating effect on the relationship between the ability of the doctor who answered first/reward and the following doctors’ participation in crowdsourced health care information websites. Thus, our third hypothesis was divided into 2 categories as follows.

1. Central route processing: The question’s complexity has a positive moderating effect on the relationship between the ability of the doctor who answered first and the following doctors’ participation in crowdsourced health care information websites.
2. Peripheral route processing: The question’s complexity has a negative moderating effect on the relationship between the reward and the following doctors’ participation in crowdsourced health care information websites.

**Figure 1:**
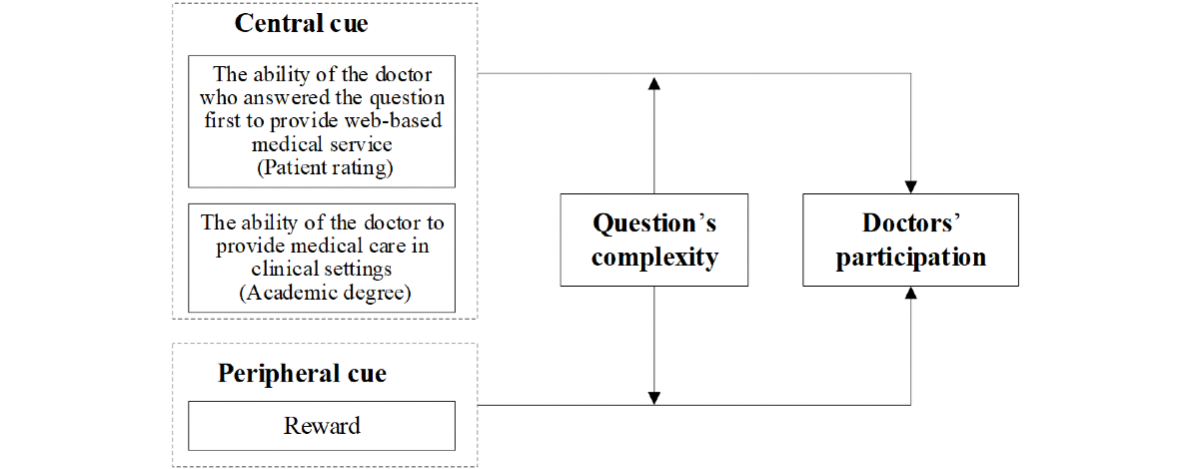
The research framework.

Based on the above hypotheses, the research framework is shown in Figure 1.

## Methods

### Research Context

**Figure 2:**
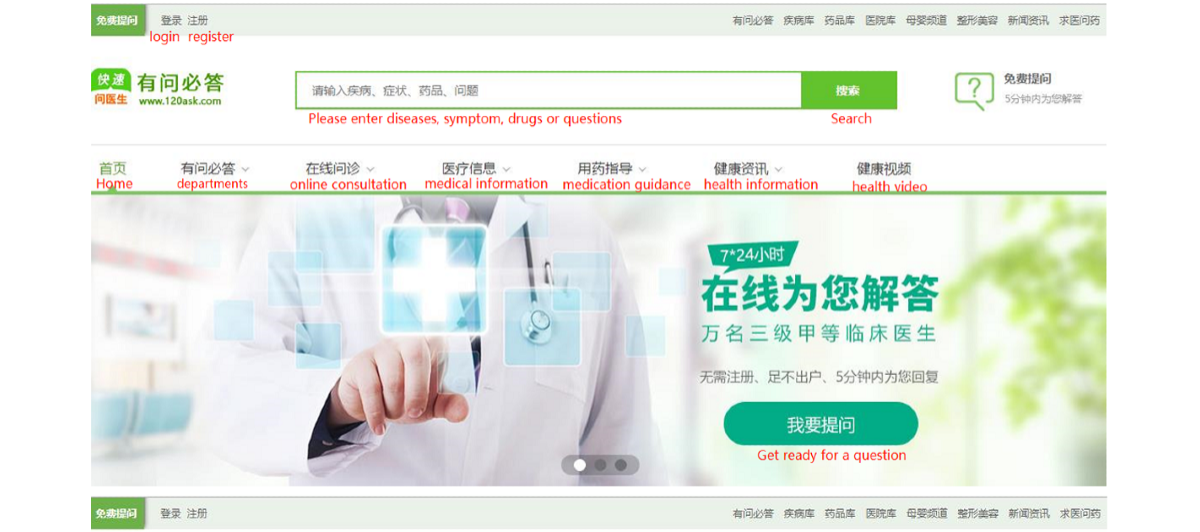
The 120ask website.

**Figure 3:**
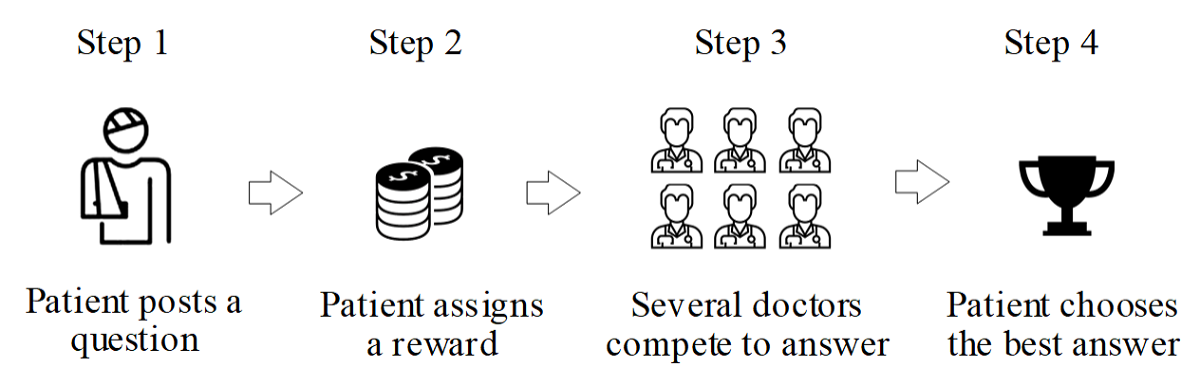
Process of medical service provision in a crowdsourced health care information website.

The 120ask website (www.120ask.com, see Figure 2) was used to obtain empirical results in this study. This website was established in 2004, and it provided a community for patients and doctors in China. The 120ask website has gathered about 5 million qualified doctors and more than 264 million patients. On this website, thousands of new health-related questions are received each day. The 120ask website is one of the top online health community platforms in China, and the monthly number of active numbers remain at above 40 million people [60]. In this platform, the doctors can share knowledge and information about the diseases and help patients improve their health conditions and receive medical diagnoses quickly and conveniently. Crowdsourced health care information websites have 2 groups of users: patients and doctors. These services allow patients to ask a health-related question to undefined doctors. The process of the crowdsourced health care information websites is as follows. First, the patient posts a question into the question pool in the online crowdsourced medical service platform with a reward and a time frame to reply. Second, within the restricted time, doctors can freely choose to answer or not and compete to win the best answer, as the reward would be given for the best answer. Third, the best answer is selected by the patient, and the corresponding doctor is granted the reward. The process of medical service provision in a crowdsourced health care information website is shown in Figure 3.

**Figure 4:**
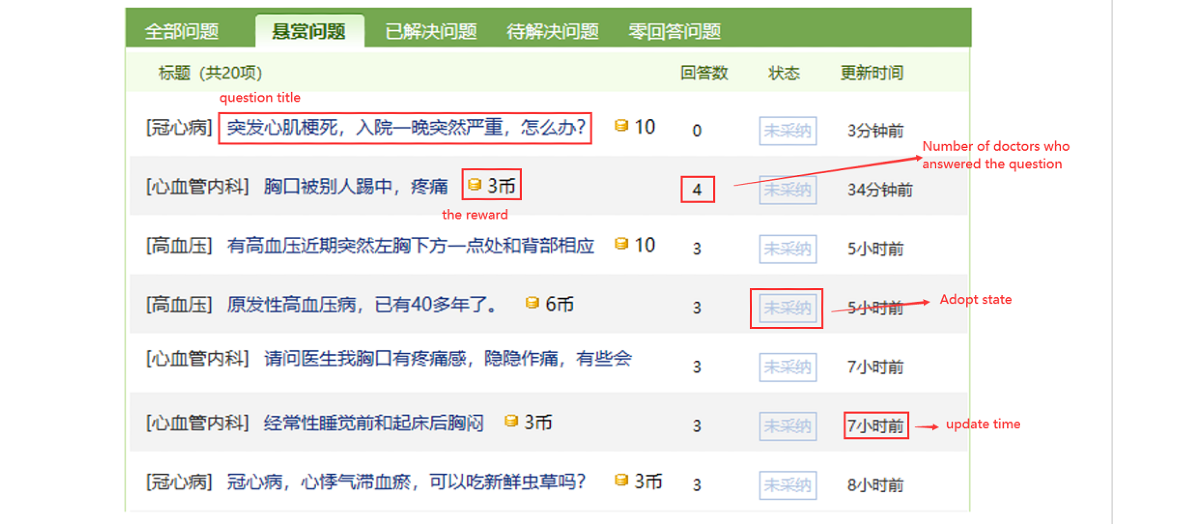
Parts of the history of the records in the crowdsourced health care information website.

We chose the 120ask website to conduct our empirical study for the following reasons. First, the website has the history of all the consultation records saved that would help patients seek health information about similar diseases (Figure 4). Second, consultation records this website are public, which provides the patient with basic information such as gender and age. Third, the doctor’s information is available on the website. Fourth, it has a large number of registered users, which enables this website to process data that are under private protection. The above features make the 120ask website a fundamentally useful website for our study.

### Sample and Data Collection

We collected the patients’ health-related questions on the crowdsourced health care information websites, and doctors provided their suggestions or advice in these websites. To examine the complete interaction process between the patient and the doctor, we chose questions that were already assigned as the best answer by the patients. We wrote a crawler in Python to download data in the crowdsourced health care information websites. For each user, we built a list of historical information, including the questions, answers, participating user identification numbers, and other features. The data were cleaned in advance by removing meaningless characters such as repeated characters and unanswered questions in text queries. Finally, 1524 complete interact process records were identified with 3245 answers in 2014-2015, and these were included in the empirical study.

### Variables and Model Estimation

We tested our hypotheses by using the ordinary least squares (OLS) model. In our research, we chose the ability of the doctor who answered first as the cue of the central route and the patient’s reward as the peripheral route information. We divided the abilities of the doctors into 2 categories: web-based ability (which refers to the average score given to the doctors by the patients based on their web-based medical service quality) and clinical ability (the professional title of the doctors in the hospital). Moreover, the question’s complexity was included as the moderating variable.

#### Dependent Variables

1. Doctors’ participation (D_Participation): For each question i , the number of doctors who answered was collected and its log value was used in the models. For each question, the doctors’ answers were sorted by the answered date, and we captured all the service information about the doctor who answered first.

#### Independent Variables

##### Central Route Information

**Figure 5:**
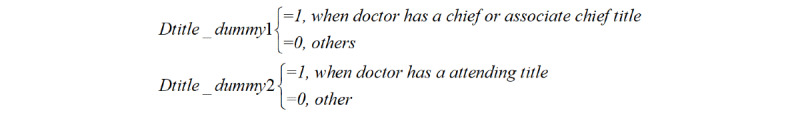
Central route information.

1. Title of the doctor who answered first (Dtitle_dummy): In China, doctors have titles that are evaluated by the government and the titles represents their clinical level of service in the hospitals; the different titles include the chief doctor, associate chief doctor, attending doctor, and others. We used 2 dummy variables to measure the doctors’ titles: Dtitle_dummy1 and Dtitle_dummy2 (Figure 5).

2. Web-based ability of the doctor who answered first (D_Score): After receiving the doctors’ web-based service, the patients can rate the doctors for the quality of their services, which ranges from 0 to 5 on the 120ask website.

##### Peripheral Route Information

1. Reward assigned by the patient (P_Reward): Patients need to set a reward for their questions. According to the rule of the 120ask website, the maximum setting value of the reward is 100 CNY (1CNY= US $0.14).

#### Moderating Effects

1. Complexity of the patient’s question (Q _Complexity): We used the length of the first doctor’s reply to measure the complexity of the patient’s question.

#### Control Variables

In our model, we also included other variables that could affect the doctors’ behavior, namely, (1) patient’s age (P_Age), (2) patient’s gender (P_Gender), (3) time limit (P_ Deadline), (4) the response speed of the doctor who answered first (D_Reponse Speed), and (5) the total number of questions answered by the doctor who answered first (D_Assistance Number). We used these variables in our research model to control the effects of the 2 different routes on the doctors’ participation. All variables and their descriptions are shown in Table 1.

**Table 1:** Description of the variables.

Variables		Variable symbol	Description
**Dependent variable**			
	Doctors’ participation	D_Participation	The number of doctors who answered the question *i*. Its log value is used in the models.
**Independent variables**			
	Central route: the professional title of the doctor who answered first	Dtitle_dummy1	The professional titles of the doctors represent their clinical abilities. Two dummy variables are used to measure doctor titles.
		Dtitle_dummy2	
	Central route: the web-based rating of the doctor who answered first	D_Score	The score that patients rate on the doctor’s quality of medical services, which ranges from 0 to 5.
	Peripheral route: reward	P_Reward	The reward that the patient assigns to the question *i*. The maximum value is 100 CNY^a^.
**Moderating effect**			
	Question’s complexity	Q _Complexity	The number of characters in the first doctor’s response is used to measure the complexity of the question that the patient posts. Its log value is used in the models.
**Control variables**			
	Patient’s age	P_Age	Its log value is used in the models.
	Patient’s gender	P_Gender	1 for male and 0 for female.
	Time limit	P_ Deadline	The time limit that the patient sets to the questions. Its log value is used in the models.
	Response speed of the doctor who answered first	D_ Response speed	The response speed of the doctor who answered first is included in the model.
	Total number of questions by the doctor who answered first	D_ Assistance numbers	The total number of questions that the doctor has answered no matter whether he/she has received a reward.

^a^1CNY= US $0.14

### Model

The empirical model is as follows:

Ln(D_Participants)=β_0_ + β_1_P_Gender + β_2_Ln(P_Age) + β_3_Ln(P_Deadline) + β_4_Ln(D_Reponse speed) + β_5_D_Assistance + β_online_P_Reward + β_offline1_Dtitle_dummy1 + β_offline2_Dtitle_dummy2 + β_6_D_Score + β_7_Ln(Q_Complexity) + β_8_P_Rewards×Ln(Q_Complexity) + β_9_Dtitle_dummy1×Ln(Q_Complexity) + β_10_Dtitle_dummy2×Ln(Q_Complexity) + β_11_D_Score×Ln(Q_Complexity) + ε_0_

### Empirical Results

The summary of the statistics of our main variables and their correlations are presented in Table 2. All variables were correlated with the doctors’ participation, except the patient’s age and the total number of answers of the doctor who answered first. Meanwhile, the correlations between the independent variables and the control variables were low, which enabled us to obtain stable results.

The empirical results are shown in Table 3 hierarchically. The results for the model with the control variables are shown in Model 1, and then the independent variables, the moderating variable, and the interaction terms in Models 2-4 were added. The adjusted *R*^2^ (>25%) and *F* values were reasonable and significant. All the variance inflation factor statistics for the variables were less than 2.0, which indicated the absence of multicollinearity.

We include control variables, that is, P_Gender, P_Age, P_Deadline, D_Response Speed, and D_Assistance Number, to address the potential endogenous issue. The results showed that all the control variables have correlations with doctor participation except P_Age and D_Assistance number. Our results revealed that when the poster (patient) is a male, the doctors’ participation will increase (β=.114, *P*<.001). A longer deadline improves the doctors’ participation significantly (β=.023, *P=.*006). Meanwhile, we also found that the response speed of the doctor who answered first positively influenced the following doctors’ participation (β=.088, *P*<.001).

The central route hypothesis predicted that the ability of the doctor who answered first would have a positive effect on the following doctors’ participation in the crowdsourced health care information websites. Based on the empirical results, we found that both web-based ability and the professional title of the doctor who answered first positively influenced the following doctors’ participation, and the central route hypothesis was supported. With regard to the professional title of the doctor who answered first (in Model 2 of Table 3), the coefficients of Dtitle_dummy1 (β_offline1_=.177, *P*<.001) and Dtitle_dummy2 (β_offline2_=.063, *P=.*048) were positive and statistically significant. With regard to the professional title of the doctor who answered first (in Model 2 of Table 3), the coefficient of D_Score (β_online_=.418, *P*<.001) was found to be positive and statistically significant. The peripheral cue hypothesis predicted that the reward has a positive effect on the doctors’ participation in crowdsourced health care information websites. Based on the results in Table 3, we find that the peripheral cue hypothesis is supported based on the coefficient of the reward (β=.019, *P*<.001). Our model suggests that the question’s complexity has a moderating effect on the relationships between the central route processing/peripheral route processing and doctors’ participation. In Table 3, we find that the web-based ability of the doctor who answered first has a significant moderating effect (β=.186, *P=.*05), but the moderating effect of the professional titles of the doctors is not significant. We also obtained the opposite direction of the moderating effects of reward (β=–.0003, *P=.*10). Therefore, central route processing is partly supported and peripheral route processing is not supported.

**Table 2:** Descriptive statistics and correlations of the variables.

Variable, mean (SD)		D_Participation	P_Gender	P_Age	P_ Deadline	D_Reponse Speed	D_Assistance Number	P_Rewards	Dtitle_dummy1	Dtitle_dummy2	D_Score	Q_Complexity
**D_Participation**, 2.16 (1.228)												
	*r*	1	0.114	–0.002	0.044	–0.037	0.294	0.341	0.183	–0.110	0.258	0.067
	*P* value	—^a^	<.001	.94	.08	.15	<.001	<.001	<.001	<.001	<.001	.01
**P_Gender**, 0.46 (0.499)												
	*r*	0.114	1	0.002	0.040	0.032	0.025	0.124	0.016	0	0.037	–0.021
	*P* value	<.001	—	.95	.12	.22	.34	<.001	.54	.99	.15	.43
**P_Age**, 29.89 (16.62)												
	*r*	–0.002	0.002	1	–0.008	–0.004	–0.031	0.040	–0.035	–0.008	0.011	–0.058
	*P* value	.93	.95	—	.76	.89	.23	.13	.17	.75	.67	.02
**P_ Deadline**, 2.16 (1.228), 0.46 (0.499)												
	*r*	0.044	0.040	–0.008	1	0.119	–0.039	–0.021	0.003	–0.151	0.120	0.144
	*P* value	.08	.12	.76	—	<.001	.13	.41	.91	<.001	<.001	<.001
**D_Reponse Speed**, 173.50 (1457.6)												
	*r*	–0.037	0.032	0	0.119	1	–0.052	–0.006	–0.014	0.009	–0.036	0.007
	*P* value	.15	.22	.89	<.001	—	.04	.83	.59	.74	.16	.79
**D_Assistance Number**, 154.55 (122.6)												
	*r*	0.294	0.025	–0.031	–0.039	–0.052	1	0.059	0.113	–0.129	0.295	0.008
	*P* value	<.001	.34	.23	.13	.04	—	.02	<.001	<.001	<.001	.76
**P_Rewards**, 5.37 (8.76)												
	*r*	0.341	0.124	0.040	–0.021	–0.006	0.059	1	0.020	0.032	0.025	0.168
	*P* value	<.001	<.001	.13	.41	.83	.02	—	.44	.21	.34	<.001
**Dtitle_dummy1**, 0.26 (0.440)												
	*r*	0.183	0.016	–0.035	0.003	–0.014	0.113	0.020	1	–0.544	0.278	0.056
	*P* value	<.001	.54	.17	.91	.59	<.001	.44	—	<.001	<.001	.03
**Dtitle_dummy2**, 0.45 (0.498)												
	*r*	–0.110	0	–0.008	–0.151	0.009	–0.129	0.032	–0.544	1	–0.370	–0.018
	*P* value	<.001	.99	.75	<.001	.74	<.001	.21	<.001	—	<.001	.496
**D_Score**, 4.67 (0.201)												
	*r*	0.258	0.037	0.011	0.120	–0.036	0.295	0.025	0.278	–0.370	1	0.017
	*P* value	<.001	.15	.67	<.001	.16	<.001	.34	<.001	<.001	—	.52
**Q_Complexity**, 88.71 (64.48)												
	*r*	0.067	–0.021	–0.058	0.144	0.007	0.008	0.168	0.056	–0.018	0.017	1
	*P* value	.01	.43	.02	<.001	.79	.76	<.001	.03	.496	.52	—

^a^Not applicable.

**Table 3:** Empirical model results.

Variables	Model 1^a^		Model 2^b^		Model 3^c^		Model 4^d^	
	β (SD)	*P* value	β (SD)	*P* value	β (SD)	*P* value	β (SD)	*P* value
P_Gender	.114 (.027)	<.001	.065 (.025)	.007	.065 (.025)	.008	.065 (.025)	.007
P_Age	.012 (.016)	.35	.015 (.015)	.32	.016 (.015)	.32	.015 (.015)	.27
P_ Deadline	.023 (.009)	<.001	.017 (.008)	.05	.021 (.008)	<.001	.023 (.008)	<.001
D_Response Speed	.088 (.007)	<.001	.067 (.007)	<.001	.064 (.007)	<.001	.065 (.007)	<.001
D_Assistance Number	–.035 (.024)	.22	–.025 (.022)	.32	–.022 (.022)	.32	–.19 (.022)	.28
P_Rewards	—^e^	—	.019 (.001)	<.001	.018 (.001)	<.001	.030 (.007)	<.001
Dtitle_dummy1	—	—	.177 (.034)	<.001	.186 (.034)	<.001	.307 (.173)	.06
Dtitle_dummy2	—	—	.063 (.032)	.05	.066 (.032)	.005	–.066 (.160)	.57
D_Score	—	—	.418 (.070)	<.001	.328 (.073)	<.001	–.386 (.307)	.11
Q_Complexity	—	—	—	—	.057 (.015)	<.001	–.807 (.368)	.049
P_Rewards×Q_Complexity	—	—	—	—	—	—	–.006 (.002)	<.001
Dtitle_dummy1×Q_Complexity	—	—	—	—	—	—	–.044 (.041)	.28
Dtitle_dummy2×Q_Complexity	—	—	—	—	—	—	.011 (.037)	.76
D_Score×Q_Complexity	—	—	—	—	—	—	.186 (.078)	.07

^a^Adjusted *R*^2^: 0.104 ; *F* change: 34.083 (*P*<.001).

^b^Adjusted *R*^2^: 0.244; *F* change: 66.852 (*P*<.001).

^c^Adjusted *R*^2^: 0.251; *F* change: 14.030 (*P*<.001).

^d^Adjusted *R*^2^: 0.253; *F* change: 2.076 (*P*=.08).

^e^Not available.

### Robustness Check

To check the robustness of our results, we chose questions with a deadline of less than 41 days (the average value of P_ Deadline) as our sample. Finally, 1301 doctors were included in the model. A long deadline may reduce the doctors’ enthusiasm to answer the questions as the payback time is unpredictable. In addition, the patients’ sincerity may be questioned when they post questions with a long deadline. Table 4 presents the results of our model robustness, which was estimated using OLS. The results are consistent with our main findings, and our empirical results were found to be robust.

**Table 4:** Robustness check.

Variables	Model 1^a^		Model 2^b^		Model 3^c^			Model 4^d^	
	β (SD)	*P* value	β (SD)	*P* value	β (SD)	*P* value	β (SD)		*P* value
P_Gender	.117 (.028)	<.001	.072 (.026)	<.001	.073(.026)	<.001	.072 (.026)		.002
P_Age	.012 (.016)	.42	.015 (.015)	.39	.016(.015)	.40	.016 (.015)		.30
P_ Deadline	.022 (.011)	.02	.012 (.010)	.27	.021(.010)	.05	.023 (.010)		.003
D_Response Speed	.088 (.008)	<.001	.067 (.007)	<.001	.065(.007)	<.001	.066 (.007)		<.001
D_Assistance Number	–.038 (.024)	.17	–.026 (.022)	.36	–.024(.022)	.36	–.21(.022)		.35
P_Rewards	—^e^	—	.019 (.001)	<.001	.018(.001)	<.001	.031(.007)		<.001
Dtitle_dummy1	—	—	.180 (.035)	<.001	.192(.035)	<.001	.328 (.175)		.009
Dtitle_dummy2	—	—	.068 (.032)	.02	.072 (.032)	.02	–.070 (.162)		.66
D_Score	—	—	.414 (.070)	<.001	.321(.074)	<.001	–.393 (.310)		.13
Q_Complexity	—	—	—	—	.058(.015)	<.001	–.803 (.371)		<.001
P_Rewards×Q_Complexity	—	—	—	—	—	—	–.007 (.002)		<.001
Dtitle_dummy1×Q_Complexity	—	—	—	—	—	—	–.033 (.040)		.14
Dtitle_dummy2×Q_Complexity	—	—	—	—	—	—	.033 (.037)		.78
D_Score×Q_Complexity	—	—	—	—	—	—	.186 (.078)		.04

^a^Adjusted *R*^2^: 0.105 ; *F* change: 33.492 (*P*<.001).

^b^Adjusted *R*^2^: 0.244; *F* change: 64.256 (*P*<.001).

^c^Adjusted *R*^2^: 0.251; *F* change: 13.692 (*P*<.001).

^d^Adjusted *R*^2^: 0.253; *F* change: 2.179 (*P*=.09).

^e^Not available.

## Discussion

### Principal Findings

Overall, our results provide us with valuable insights into the role of the central and peripheral cues in crowdsourced health care information websites based on the framework of ELM. Our statistical evidence suggests that the following doctors’ participation is related to the ability of the doctor who answered first. Based on the ELM, the central cues present the information needed for in-depth thinking. The ability of the doctor who answered first was used as the central cue in our study. In crowdsourced health care information websites, doctors could read the information in the prior answers before they answered the question. Based on the signal theory [61], we believe that the ability of the doctor who answered first can reflect the question’s value and lead to a positive behavioral implication and increase the participation intention of the other doctors. Therefore, highly competent doctors should play a leading role in solving health problems.

The results relating to the rewards posted by the patients indicated that this variable is closely related to the intention of the doctors’ participation. Our results suggest that similar to other crowdsourcing fields [25,51], doctors are very concerned about the reward. The reward drives doctors to participate in services that help patients solve their health problems. We also believe that more doctors will participate in providing medical services through crowdsourced health care information websites when the reward is higher than they expected. Therefore, setting a high reward can increase the participation of a large number of doctors to answer the question.

Our empirical results show that there is a significant moderating effect between the question complexity and the dual route. For the central route, our results show that the question’s complexity can enhance the effect of the ability of the doctor who answered first on the following doctors’ participation. Questions with high complexity are often more worthy for doctors with high competencies to answer, and our results suggest that highly competent doctors should take the responsibility to solve questions with high complexity. However, we found that question complexity does not have a significant moderating effect. A possible reason is that the doctor’s web-based ability (the average score rated by the patients) represents the doctor’s comprehensive web-based ability, which is more effective than the professional title in crowdsourced health care information websites because the entire interaction process is performed on the internet. Another reason is that the active group of doctors in crowdsourced health care information websites is mostly middle-level doctors who are younger and have more time to help patients in web-based medical services. For the peripheral route, we found that the reward was not very important when the question complexity was high because of the doctor’s altruism, which has also been verified in other online health communities [29]. Therefore, the question complexity positively moderates the influence of the ability of the first responding doctor on the following doctors’ participation and negatively moderates the influence of the reward factor on the following doctors’ participation.

### Contributions of This Study

This study has made the following contributions. First, to our knowledge, we are among the first to extend the ELM model to crowdsourced health care information websites. Previous studies have often used ELM in consumer adoption or satisfaction, information technology adoption, information adoption, and other areas [62-64]. We extended these previous studies by using ELM to investigate the doctors’ participation in providing medical services through crowdsourced health care information websites and explored the different routes of different cues for understanding the behaviors of doctors’ participation in crowdsourced health care information websites. Moreover, we used the question’s complexity to investigate the moderating effects on the roles of the 2 routes. Second, our study has added valuable information to the existing studies on online health communities. The previous studies mainly focused on one-to-one consultant service to study the satisfaction of the patient or to study the relationship between the signals of other doctors (eg, price and reputation) and patient’s choice [38,65]. Our study investigated the doctors’ participation in one-to-crowd crowdsourced health care information websites. Third, we focused on expert-based question-and-answer websites, whereas the existing studies are based on Baidu Zhidao and Wiki Answers, which belongs to the ordinary community-based question-and-answer websites [66,67]. Our study broadens the research on expert-based question-and-answer websites, especially in the medical domain.

Our research has three major implications for practice. First, we found that the behaviors of the doctors involved in answering the patient’s questions are influenced by the behavior of the ability of the doctor who first responded to the patient’s question. Therefore, competent doctors should be encouraged to take up the leadership positions in online health communities and be actively involved in crowdsourced health care information websites. Second, according to our results, if patients want to receive more answers, they should increase the rewards for the question or invite highly competent doctors to answer the questions. Third, if the managers of the online health communities want to operate the platform successfully and make a profit, they should encourage doctors by providing an incentive mechanism to answer the question quickly and thoughtfully, as shown in our results.

### Limitations of This Study

This study had the following limitations. First, this study selected only 1 online health community to investigate the participation behaviors of the doctors. Future studies should select different online health communities to compare the differences. Second, future research should consider other types of questions of the patients, such as questions related to emotional support needs or professional health care needs. Third, future research should adopt a longitudinal perspective to overcome the disadvantages of the cross-sectional data and explore the dynamics in the relationships as well.

### Conclusion

This research explored the effects of the ability of the doctor who answered patients’ questions first as well as the effects of rewards on the following doctors’ participation in crowdsourced health care information websites. We also investigated the moderating effects of the question’s complexity on these relationships. We developed a mathematical model to test our hypotheses. The empirical results supported most of our hypotheses. This study can help academicians to better understand the evaluation and the decision processes used by doctors when considering the web-based health-related crowdsourcing services. Moreover, this study has provided several implications for the practice of online health community managers and users.

## Abbreviations

ELM: elaboration-likelihood model
OLS: ordinary least squares
